# Retinoblastoma survival trend: a 30-year analysis from a referral single center in Iran

**DOI:** 10.1186/s40942-025-00702-4

**Published:** 2025-10-17

**Authors:** Masood Naseripour, Ali Aghajani, Hengameh Kasraei, Reza Mirshahi, Ahad Sedaghat, Parya Abdolalizadeh, Mohammadreza Fazel, Samira Chaibakhsh

**Affiliations:** 1 Finetech in Medicine Research Center, Iran University of Medical Sciences, Tehran, Iran; 2 Department of Ophthalmology, Eye Research Center, The Five Senses Health Institute, Moheb Kowsar Hospital, School of Medicine, Ira University of Medical Sciences, Tehran, Iran; 3 Isfahan eye research center, Department of Ophthalmology, Isfahan University of medical sciences, Isfahan, Iran; 4 Cardiovascular Epidemiology Research Center, Rajaie Cardiovascular Institute, Tehran, Iran; 5 School of Medicine, Urmia University of Medical Science, Urmia, Iran

**Keywords:** Retinoblastoma, Mortality, Trend, Childhood, Tumor

## Abstract

**Background:**

The treatment of retinoblastoma (Rb) has undergone significant improvements over the last century. This study aims to assess the trend of enucleation and mortality in retinoblastoma patients during the last 3 decades.

**Methods:**

The study utilized data from the referral center for ocular oncology in Rasool Akram Hospital, Tehran, Iran. It included all patients diagnosed with Rb from August 1991 to December 2018. The study investigated the trend of enucleation and mortality during three-time intervals: before 2001 (T1), during 2001–2007 (T2), and 2008–2018 (T3). Additionally, it assessed the trend of enucleation and mortality based on laterality, age and presentation of Rb (strabismus and leukocoria).

**Results:**

The incidence of enucleation decreased significantly from T1 to T3 (74–41%) during the study period (p-value < 0.001). Pairwise comparisons between T1 and T3 revealed a significant decrease in the incidence of enucleation (74% vs. 41%, p-value < 0.001). The study also demonstrated a significant reduction in the incidence of enucleation when comparing T2 to T3 (60% vs. 41%, p-value < 0.001). Comparing time intervals, there was no significant difference between T2 and T3 regarding the incidence of death (4% vs. 1%), but both intervals had statistically significant lower death rates compared with T1 (26%, both p-values < 0.001).

**Conclusion:**

This study revealed that Introduction of systemic chemotherapy as mainstay of Rb treatment, has led to a significant reduction in mortality and morbidity rates. The incorporation of targeted chemotherapy has further decreased the need for enucleation, but it has not substantially impacted the mortality rate. Unfortunately, in spite of reduction trend in enucleation, systemic and targeted chemotherapy was unable to save the affected globe in nearly half of the patients, even when the malignancy was diagnosed in the earlier stages.

## Background

Retinoblastoma (Rb) is the most common eye malignancy in childhood globally, comprising 2.5–4% of all pediatric cancers. The estimated incidence worldwide is approximately 1 in 15,000 to 20,000 live births with significant variation in its prevalence among different regions and countries [1, 2].

Early diagnosis of retinoblastoma is vital due to its early onset and distinct visible symptoms, such as leukocoria or strabismus. This enables timely intervention, significantly improving the chance of successful treatment and vision preservation [3].

The main goal of retinoblastoma treatment is to save the children’s lives, followed by efforts to preserve the affected eye and the vision whenever possible. Over the last few decades, the treatment options have considerably evolved and now include systemic chemotherapy, radiation therapy, cryotherapy, laser therapy, intravitreal chemotherapy, intra-arterial chemotherapy, or in severe cases, enucleation [4]. During the 1990s, enucleation was the only available approach to encounter the tumor and it was commonly successful in ensuring the patient’s survival [5]. In the early 2000s, systemic chemotherapy with vincristine, etoposide and carboplatin (VEC regimen or high-dose VEC Regimen) was the main treatment option to approach the tumor. The most concerning adverse event was that after implementing these regimens children were subjected to toxic doses of chemotherapy medications due to the chemo-resistant nature of retinoblastoma. The Introduction of targeted chemotherapy including intra-arterial chemotherapy (IAC) with agents such as melphalan, topotecan and carboplatin as well as intravitreal injection of these drugs, was first pioneered by Japanese researchers [6]. Later revised and complemented by American teams in 2008, this treatment modality represented another significant milestone in management of retinoblastoma. This innovative approach allows higher drug concentrations to be delivered directly to the tumor site, minimizing systemic side effects and enhancing therapeutic efficacy [7, 8]. These therapeutic advancements coupled with early detection have significantly improved survival rates and the quality of life for affected children over decades [9].

Trends in retinoblastoma survival rates have varied across different studies and regions. For instance, researchers in Thailand reported an upward trajectory from 1990 to 2009 [10]. Similarly Warda et al. documented an increase in 5-year survival rate, rising from 85 to 98% between 1960 and 2022 [11]. In contrast, other studies using the Surveillance, Epidemiology, and End Results (SEER) database revealed a decline in retinoblastoma survival rates [12].

Research on the trend of retinoblastoma can guide public health strategies, preventive measures, and treatment approaches to effectively improve outcomes for affected children. This study was conducted to evaluate the trends in mortality and enucleation rate over the past 3 decades in a referral center in Tehran, Iran.

## Materials and methods

This single-center, retrospective cohort study was conducted using the database of retinoblastoma patients who were diagnosed and managed in Rasool Akram Hospital, a tertiary referral center for ocular oncology, affiliated with Iran University of Medical Sciences, Tehran, Iran. All data was extracted after written informed consent was obtained from children’s parents. The research protocol was approved by the medical ethics committee of Iran University of Medical Sciences (IR.IUMS.FMD.REC.1398.292) and the study adhered to the ethical principles outlined in the Declaration of Helsinki.

We categorized the patients based on the year they were diagnosed with Rb with the rationale of significant differences in treatment options across the following time periods: T1: 1990–2000, T2: 2001–2007 and T3: 2008–2018. Primary enucleation with or without focal radiotherapy was the only treatment choice for the Rb patients before the start of 21st century (Patient assigned to T1). The mainstay treatment for Rb patients assigned to T2 was systemic chemotherapy (VEC or high dose VEC) with the administration of focal therapies including plaque radiotherapy, transpupillary thermotherapy, cryotherapy, and subconjunctival chemotherapy in specific situations. Following the introduction and availability of targeted chemotherapy, intra-arterial and intravitreal medications were added to systemic chemotherapy for Rb patients assigned to T3. Enucleation was limited to those with group E tumors or those who did not respond to first-line management after 2000 (Patients assigned to T2 and T3). The treatment selection was based on the patient’s socioeconomic status, laterality of the disease, ICRB grouping of each eye, patients’ age and ocular oncologist preference.

The extracted data included the following: Demographic factors such as age at the presentation and gender across all three time periods, total number of Rb patients and eyes affected with tumoral lesions taking into account the laterality of the disease in each time period, Total number and percentage of initial presentation including strabismus or leukocoria that led to the diagnosis of Rb in each time period, Total number and percentage of eyes that underwent either primary or secondary enucleation in each time period, Total number and percentage of the patients who unfortunately expired in each time period. Records without written informed consent or missing the above data were excluded from the final analysis.

### Statistical analysis

In this study, we investigated the trends of enucleation and mortality or survival rates during the three previously mentioned time intervals. Sub-group analysis was also performed regarding laterality (Unilateral Rb Vs. Bilateral Rb), Age group (< 2 years of age Vs. >2 years of age) and initial presentation (Strabismus Vs. leukocoria) across the three time periods. Survival rates were compared with SEER database, as well. Trends were assessed using the chi-square test for trend, with the incidences of enucleation and death considered as the outcomes. All of the analyses were performed using R 4.2.1 software, and a P-value < 0.05 is considered statistically significant.

## Results

A total of 642 eyes patients were included in this study. The number of eyes in T1 (between1990 and 2000) was126, in T2 (between 2001 and 2007) was 224, and in T3 (between 2008 and 2018) was 292.

### Trend of enucleation incidence (EI)

**Fig. 1:**
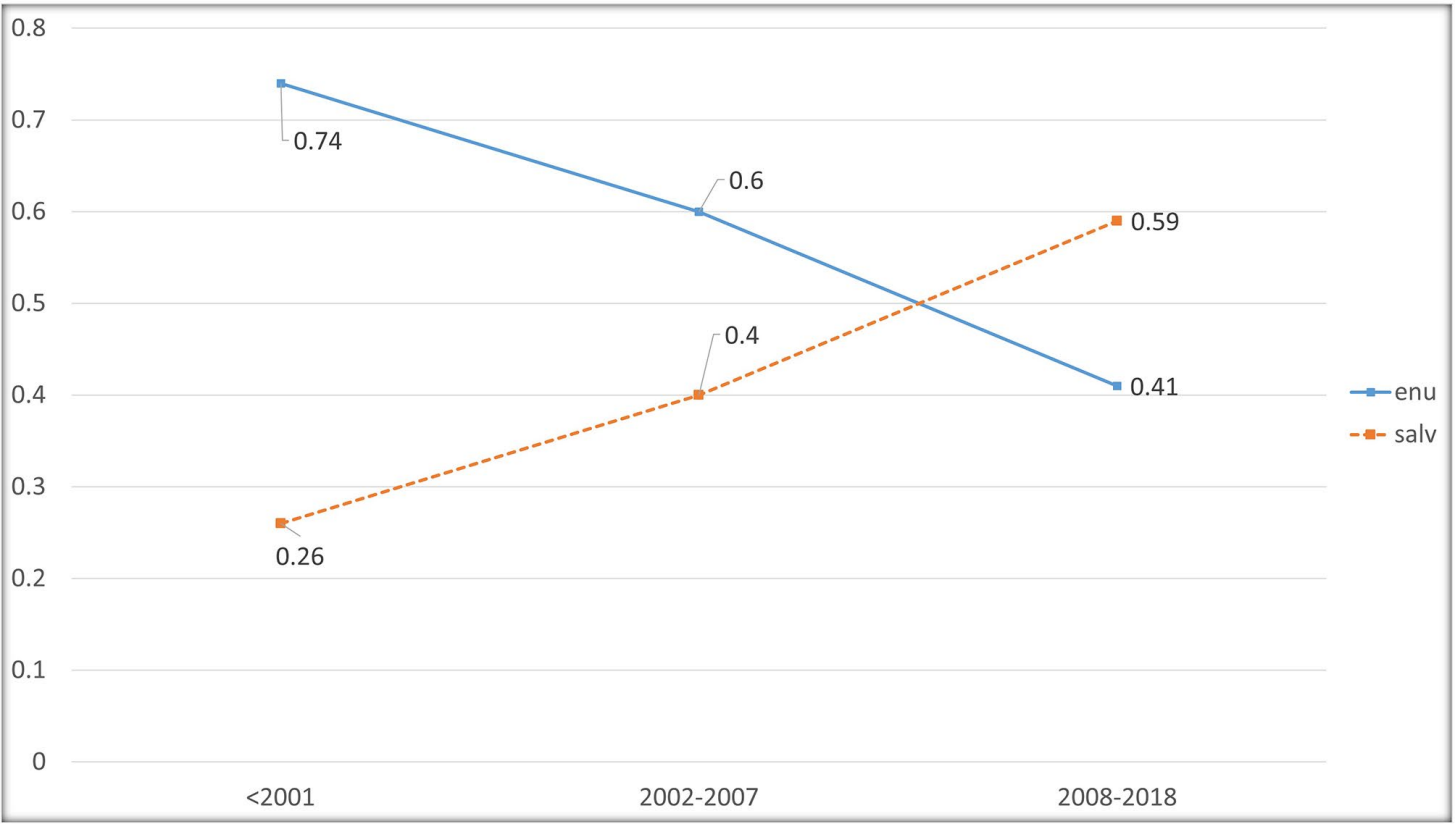
Incidence of salvaged and enucleated eyes in the study duration intervals

Figure 1 illustrates the trend of globe salvage in the three-time intervals. The incidence of enucleation decreased significantly from T1 to T3 (74–41%) during the study period (p-value < 0.001, Table 1). Pairwise comparisons between T1 and T3 revealed a significant decrease in the incidence of enucleation (74% vs. 41%, p-value < 0.001). occurrence of enucleation when comparing T2 to T3 (60% vs. 41%, p-value < 0.001). However, no significant difference was detected when comparing the incidence of enucleation between the first two-time intervals.

**Table 1:** Comparing the enucleation between the study duration intervals

Variable	< 2001	2001–2007	2008–2018	p-value
**Age**				
<=2 years	67% (44/66)	68% (64/94)	42% (58/138)	**< 0.001**
> 2 years	84% (47/56)	69% (38/55)	67% (42/63)	**0.037**
**Gender**				
Female	72% (36/50)	60% (41/68)	51% (41/81)	**0.015**
Male	75% (57/76)	75% (61/81)	49% (59/120)	**< 0.001**
**Laterality**				
Bilateral	69% (20/29)	53% (21/39)	34% (13/39)	**< 0.001**
Unilateral	76% (64/84)	83% (63/76)	63% (68/108)	**0.028**
**Leukocoria**	84% (45/53)	76% (76/100)	54% (55/102)	**< 0.001**
**Strabismus**	38% (3/8)	44% (15/34)	45% (15/33)	**0.725**

**Fig. 2:**
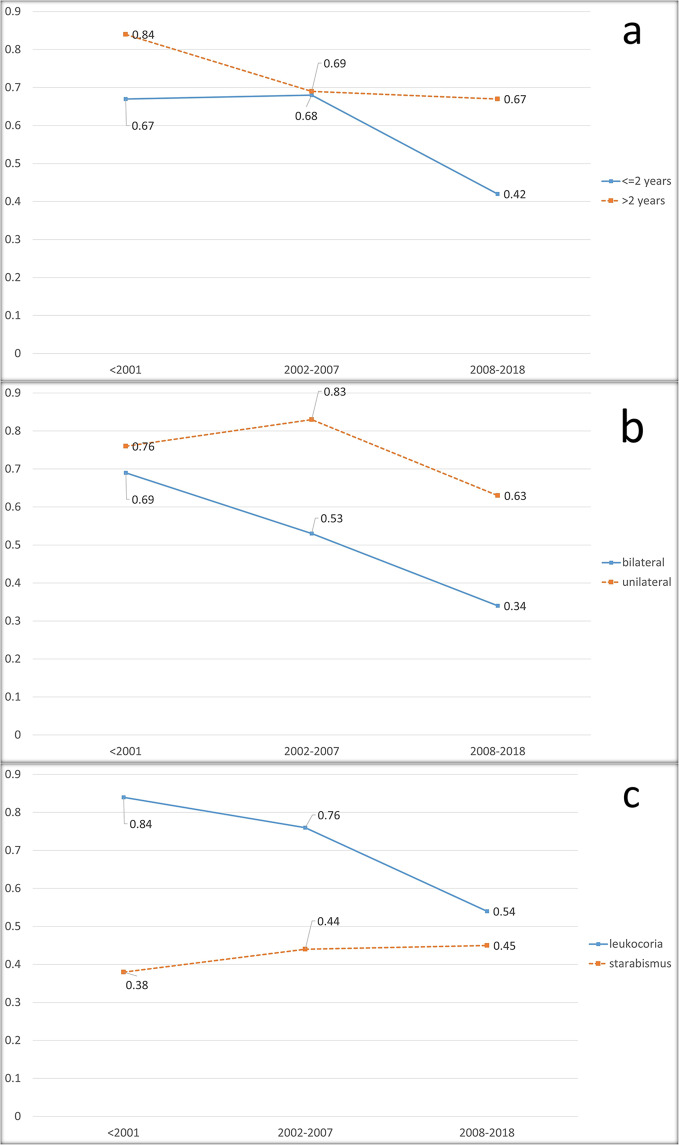
**a**: Incidence of enucleated eyes by age groups in the study duration intervals. **b**: Incidence of enucleated eyes by laterality in study duration intervals. **c**: Incidence of enucleated eyes by type of presentation in study duration intervals

Figure 2a; Table 1 depict the trend of enucleation by age across three-time intervals. The incidence of enucleation was decreased significantly from 67 to 42% during the study time in patients under 2 years of age (p-value < 0.001). Pairwise comparisons showed a significant drop from T2 to T3 (p-value < 0.001). There was not any significant difference between the incidence of enucleation between the first two-time intervals(p-value = 0.756). In patients older than 2 years, the incidence of enucleation decreased from 84% in T1 to 67% in T3 (p-value = 0.037). Although a reduction was seen when comparing the T1 to T2, the difference between them was not statistically significant (p-value = 0.062). Notably, in patients aged 2 years and above, there was a significant difference between the incidence of enucleation in T3 and T1 (67% vs. 84%, p-value = 0.032).

### Unilateral vs. bilateral

Table 1; Fig. 2b illustrate the trend of enucleation incidence by laterality. Analysis revealed a significant descending trend of enucleation in both unilateral and bilateral patients (p-value = 0.028 for unilateral and < 0.001 for bilateral). The incidence of enucleation in unilateral cases was not changed significantly when comparing T2 to T1 (p-value = 0.183) but it decreased significantly from 83% in T2 to 63% in T3 (p-value = 0.003).

Regarding bilateral patients, although the enucleation incidence was lower in T2 comparing with T1, the difference between them was not statistically significant (p-value = 0.183). The enucleation incidence in bilateral patients decreased significantly from 69% in T1 to 34% in T3 (p-value = 0.004). No significant difference was seen comparing T2 with T3 (p-value = 0.091).

### Clinical presentation

Figure 2c depicts the incidence of enucleation in patients with leukocoria and strabismus presentations. There was not any significant trend regarding enucleation incidence in patients with strabismus presentation over the study period (p-value = 0.725, Table 1). Meanwhile, the occurrence of enucleation substantially decreased from 84 to 54% throughout the study period (p-value < 0.001, Table 1) among patients whose primary presentation was Leukocoria. The pairwise comparisons showed a significant difference between the enucleation incidence of T3 and the other two-time intervals (both p-values < 0.001) in this group.

### Trend of death incidence

**Fig. 3:**
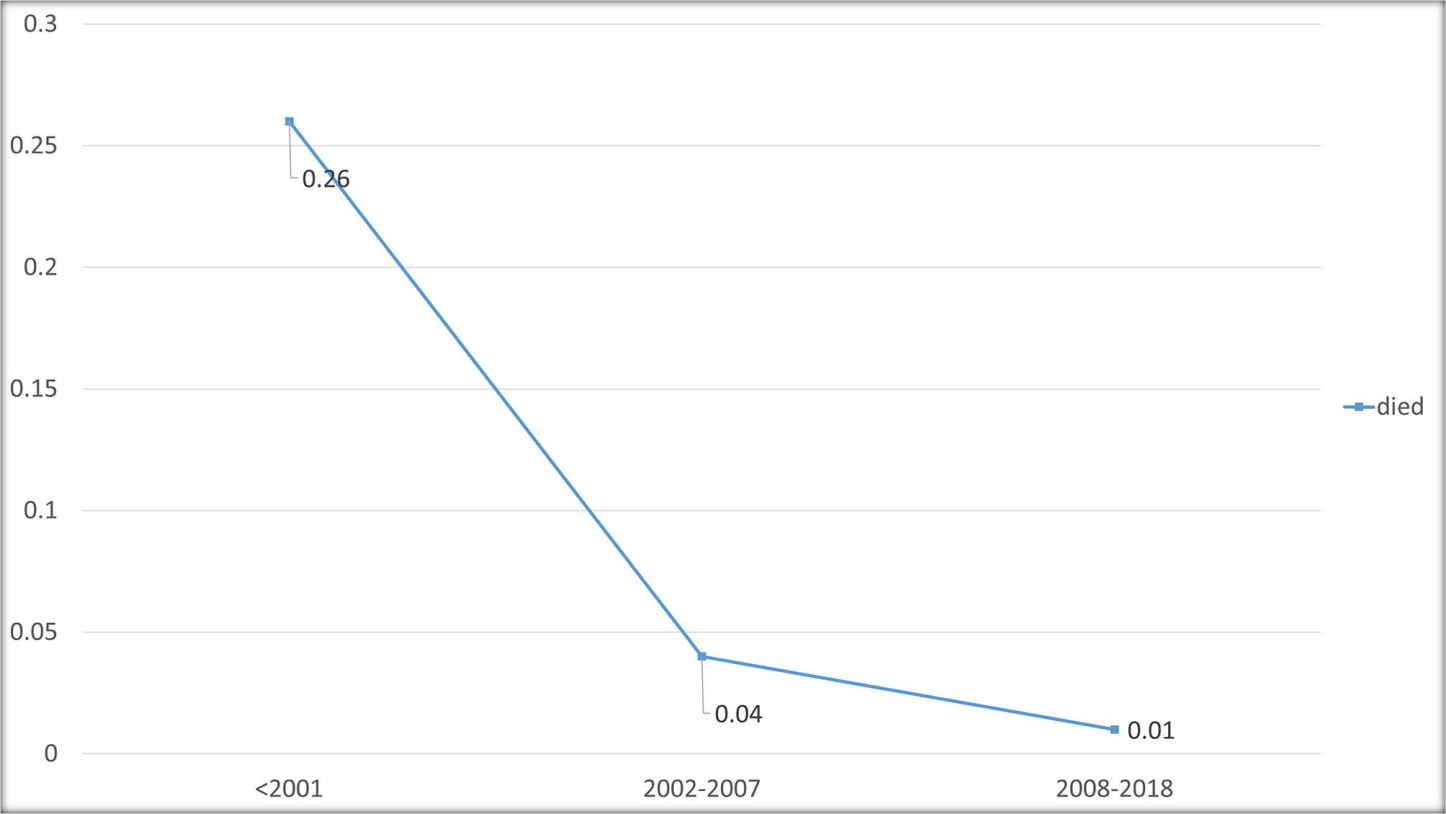
Incidence of death in the study duration intervals

Figure 3 illustrates the trend of deceased patients across the three-time intervals. A significant decrease was seen during the study period (p-value < 0.001, Table 2). When comparing time intervals, there was not any significant difference between T2 and T3 regarding the incidence of death (4% vs. 1%). However, both intervals showed lower mortality rate comparing with T1 (26%, both p-values < 0.001).

**Table 2:** Comparing the death incidence between the study duration intervals

Variable	< 2001	2001–2007	2008–2018	p-value
**Patient survival**				< 0.001
Survived	74% (92/124)	96% (143/149)	99% (198/201)	
Died	26% (32/124)	4% (6/149)	1% (3/201)	
**Age**				
<=2 years	14.3% (9/63)	5% (5/94)	2% (3/138)	**0.001**
> 2 years	38% (23/60)	1% (1/55)	0% (0/63)	**< 0.001**
**Gender**				
Female	22% (11/49)	4% (3/68)	1% (1/81)	**< 0.001**
Male	28% (21/75)	4% (3/81)	2% (2/120)	**< 0.001**
**Laterality**				
Bilateral	21% (8/38)	5% (5/73)	2% (2/93)	**< 0.001**
Unilateral	14% (10/72)	1% (1/76)	1% (1/108)	**< 0.001**
**Leukocoria**	39% (23/59)	3% (3/100)	2% (2/102)	**< 0.001**
**Strabismus**	38% (3/8)	9% (3/34)	0% (0/33)	**0.001**

**Fig. 4:**
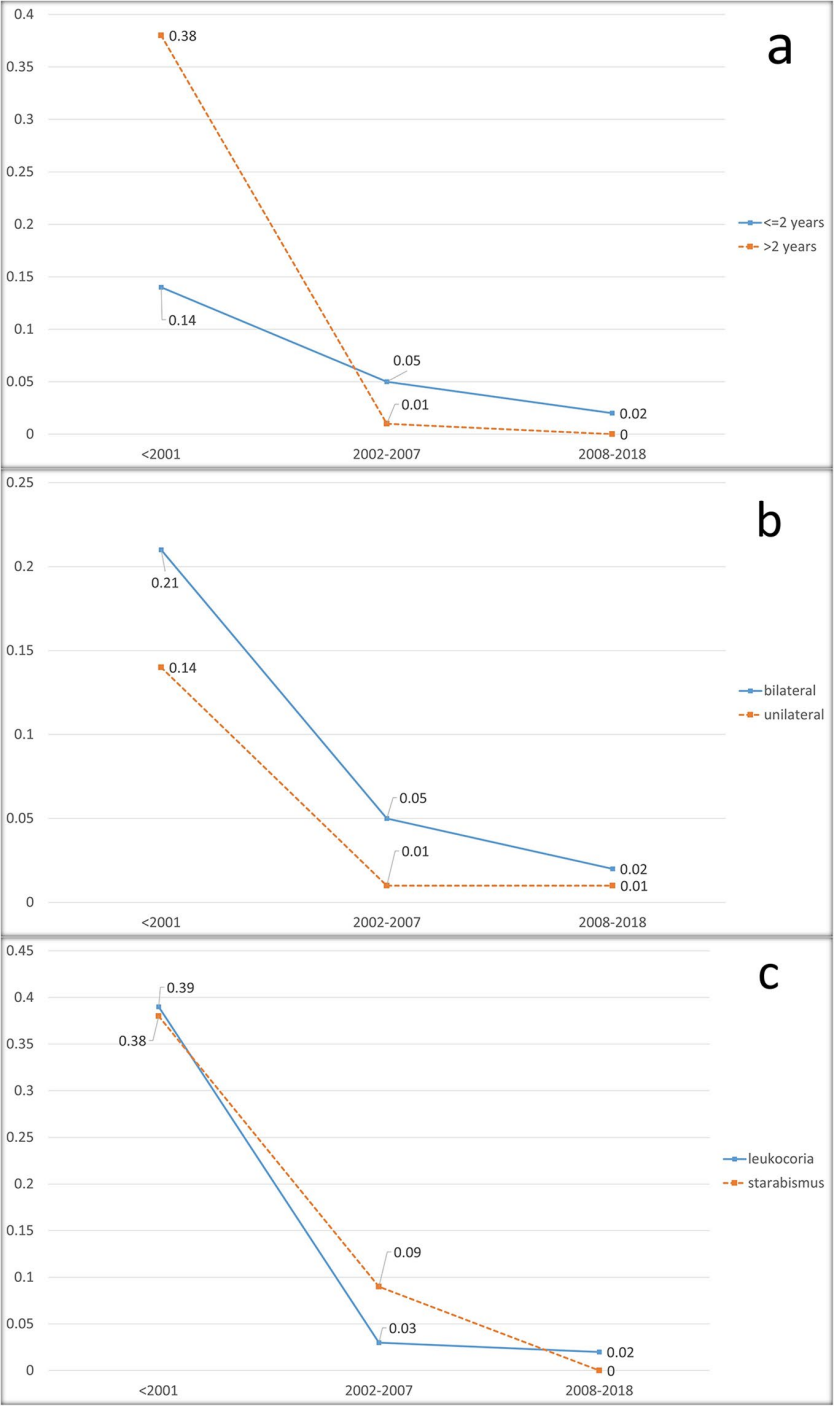
**a**: Incidence of death by age groups in the study duration intervals. **b**: Incidence of death by laterality in the study duration intervals. **c**: Incidence of death by type of presentations in study duration intervals

Figure 4a; Table 2 shows the trend of expired patients by age in the three-time intervals. Both age groups ( < = 2 and > 2 years) exhibited a significant decrease in the incidence of mortality (p-value = 0.001 and < 0.001). There was a significant difference between the incidence of death in T1 compared to T2 and T3 in both age groups (all p-values < 0.003). There was no significant difference between T2 and T3 in both age groups regarding the incidence of death (p-value = 0.897 and 0.851).

**Fig. 5:**
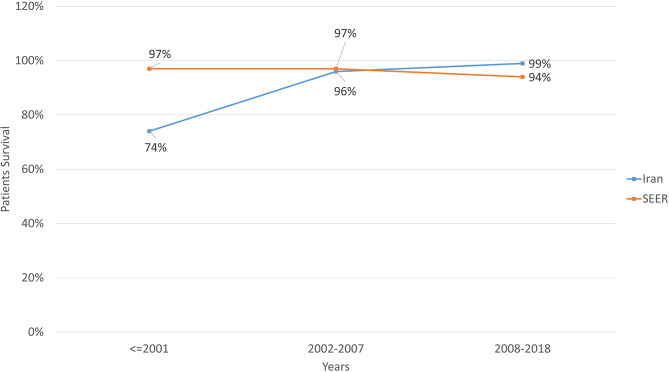
Comparing survival rates between Iran and SEER database

When comparing to SEER database in United States of America (USA) [12], patient survival showed an upward trend despite slight decrease in survival rate of US patients in recent years. (Fig. 5)

### Unilateral vs. bilateral

Figure 4b; Table 2 depicts the trend of deceased patients by laterality in the three-time intervals. Death incidence decreased significantly in both bilateral and unilateral patients (p-values < 0.001); in bilateral patients it changed from 21 to 2% and in unilateral cases this incidence decreased from 14 to 1%. Pairwise comparisons revealed that the difference in death incidence between T2 and T3 was not significant (p-values = 0.823, > 0.999) but a significant difference was detected comparing T1 to the other two intervals in both unilateral and bilateral patients (all of the p-values were < 0.001).

### Clinical presentation

Our data demonstrated that the patients with retinoblastoma initially presented with two major clinical signs, leukocoria and strabismus. We did not recognize any patient, initially presented with both leukocoria and strabismus. The incidence of death in patients with Leukocoria and Strabismus presentations has been illustrated in Fig. 4c. Death incidence decreased significantly in patients with both presentations (p-value = 0.001 and < 0.001 for strabismus and leukocoria respectively). Mortality rate in patients with leukocoria declined significantly from 39% in T1 to 3% and 2% in T2, and T3 (p-values < 0.001). There was not any significant difference between the last two intervals regarding death incidence in patients with leukocoria presentation (p-values > 0.895).

Death rate among patients with strabismus as their first presentation dropped significantly from 38% in T1 to 9 and 0% in T2, and T3 (all p-values < 0.001). There was not any significant difference between the last two intervals in terms of death incidence in patients with strabismus (p-values > 0.753).

## Discussion

The primary focus of this study is to determine the impact of systemic and targeted chemotherapy in intraocular retinoblastoma outcome in terms of both patient and globe survival. The findings reveal that although the advanced treatment modalities have resulted in significant reduction in mortality rate as well as remarkable improvement in globe preservation, these treatment options have had varying impacts on the mortality and enucleation rate.

Retinoblastoma is a potentially curable tumor with high survival rates if treated early and appropriately [13]. The World Health Organization Global Initiative for Childhood Cancer aims to raise survival for key childhood cancers, including retinoblastoma, to 60% by the year 2030 by improving health systems of low middle income countries (LMICs) [14]. Accordingly, the first aim of management of retinoblastoma is life salvage. Bothe stage of the disease at presentation and treatment modality have a significant association with survival outcomes [15]. Treatment techniques have undergone significant evolution, resulting in a notable reduction in the enucleation rate. These advancements include the use of radiotherapy, systemic and local chemotherapy, and local ablative treatments [15–18]. Consequently, the prognosis of Rb has improved dramatically and the globe preservation is considered as the second aim of Rb treatment [15]. Over the years, management goals have shifted from preserving life and the globe to maintaining a sustainable long-term vision [19, 20]. When enucleation is performed, socket rehabilitation with a primary orbital implant and customized ocular prosthesis are just as important and becomes the fourth goal of ideal management [21]. 

According to this study, the introduction of systemic chemotherapy as the cornerstone of retinoblastoma treatment has markedly reduced mortality associated with the disease (Fig. 3). This reduction in mortality rate is observed across all study subgroups; outlining the great positive impact of this treatment approach. This achievement in our study is comparable to survival rate reported from high income countries [22–24]. Unlike the data provided by SEER [12], this study observed a general consistent rise in patient survival rates. Abdelazeem et al. suggested that heroic efforts to salvage the eyes in unilateral Rb might have contributed to increasing incidence of death in their Rb population; [12] a pattern that we did not observe in our practice probably due to timely decision for enucleation. On the other hand, as illustrated in Fig. 4a, systemic chemotherapy has substantially narrowed the disparity in mortality rates between patients aged over and under two years (38% vs. 14% in T1), to the extent that the gap is no longer statistically significant. This suggests that age at presentation should not be considered a prognostic factor when systemic chemotherapy is available as a treatment option.

The treatment approach underwent revolution at two time points in Iran. The main therapeutic modalities were focal therapy, enucleation and external beam radiation therapy up to 2001 when there was a gradual policy change to apply systemic chemotherapy and focal treatments (plaque radiotherapy, transpupillary thermotherapy and cryotherapy). Intra-arterial and intravitreal chemotherapy have been launched after 2011 to increase the globe survival and preserve the vision to the fullest extent possible [25, 26]. 

No validated geographic or population preponderance for Rb incidence has been found while there is a significant disparity of survival rate among LMICs and high-income countries (HICs). It is shown that the overall patient and globe survival is largely correlated with the country’s income [24]. In HICs, the patient survival reached to nearly 100% and the therapeutic goals are emphasizing on vision preservation [27–30]. In Great Britain for the study period of 1998–2002 the 5-year survival rates was 97-100% [29]. In US over the period of 40 years the 5-year reported actuarial survival rates increased from 92.3% in 1975 to 97.3% in 2012 [27, 28]. 

On the other hand, LMICs of Asia-Pacific and Africa have the greatest disease burden owing to the large population with high birth rates [31, 32]. These countries, with 84% of global Rb children, reveal a much lower mean survival rate and their main challenges are still focusing on surviving patients and globes [14, 31–34]. The large case series of India showed 68–90% overall 5-year survival [35, 36]. The overall survival probability in Thailand and Singapore has been reported between 73 and 93% which became higher in recent years [21]. Survival rates reported from less developed countries like African countries, and Nepal are even less than 80% [33, 34, 37, 38]. In our previous reports from Iranian Rb patients, the five-year survival rate in time period of 1990 to 2001 was 70% [25]. Although it remained lower than developed countries [27–30], there was a rising trend in patient survival during 2001 to 2007 with five-year survival rate of 83% [26]. Our recent data showed that the five-year survival of Iranian patients escalated to 98.8% in 2018 is compatible with reported survival from the HICs [22]. Recent studies have demonstrated improving pattern in other LMICs like India [35]. 

Another noteworthy finding of this study is that even though adding targeted chemotherapy (intravitreal and intra-arterial) to systemic chemotherapy did not significantly improve the survival rate in any of the study subgroups, it did lead to a dramatic decrease in the total enucleation rate from 60 to 41% (Fig. 1). The subgroups in this study exhibited diverse responses to systemic and targeted chemotherapy in terms of salvaging the globe. Administering systemic chemotherapy to patients in the unilateral retinoblastoma, and under the age of two did not reduce the enucleation rate compared to conventional treatment. The reason could be that the decision to enucleating the affected eye in unilateral Rb have always been made easier (compared to bilateral Rb) and systemic chemotherapy which is mainly a means to controls and inactivates the Rb has not demonstrated sufficient localized efficacy to prompt a shift in this practice pattern.

This study shows that while choosing targeted over systemic chemotherapy has benefits like avoiding systemic complications, it does not significantly improve short-term (5-year) survival rates for patients. The early survival of Rb patients is primarily influenced by the occurrence of metastasis [12], which typically requires systemic chemotherapy. Adding local chemotherapy into the treatment regimen of the patients who have already been receiving systemic treatment did not increase the survival rate of the patients in this study. On the other hand, it is worth noting that despite successfully controlling the local disease in many patients, local chemotherapy has not been effective in preventing Rb from spreading in a subset of patients. It is essential to conduct separate studies to better understand the nature of these resistant tumors.

It is worth noting that one of the critical factors linked to the survival rate and globe salvage rate in retinoblastoma is the time taken to reach a diagnosis. Delayed diagnosis serves as an indirect measure of awareness regarding retinoblastoma and is heavily influenced by a well-organized healthcare system [13, 39]. In developing countries, lag time (time to diagnosis interval) is significantly higher than developed countries; for example it is 8 weeks in United Kingdom and 6 weeks in USA for unilateral Rb compared to 3–8 months in India [27, 29, 36, 39]. Diagnostic delay may cause the relatively advanced age of presentation in developing countries [40]. Based on recent report of retinoblastoma global study group, the mean age of Rb diagnosis is 30 months [31]. Patients from HICs with mean diagnostic age of 14 months, are diagnosed earlier than patients from MLICs with mean age at diagnosis of 30 months [31]. We also observed a trend of reduction in the age of diagnosis from 30 months during 1990–2001 and 28.5 months during 2001–2007 to 24 months during 2008–2018 among Iranian patients that suggests earlier diagnosis by time [22, 25, 26, 41]. But, it is still far more than HICs values. Diagnostic delay also leads to significant difference in the percentage of patients presenting with advanced disease among developed and developing countries; as much as 30–40% in developing nations compared to only 2–5% in developed nations [13, 31, 42, 43]. Hence, it is associated with increased mortality and extraocular Rb in developing nations [13, 15, 39]. 

In addition to improving patient outcomes and increasing survival rates, early diagnosis can also have a positive impact on the future socioeconomic status of both patients and their families. Management of retinoblastoma by focal therapy alone in the early stages rather than chemotherapy and possible radiation in the late stages will induce less economic burden.

As is shown in Fig. 2c, Systemic chemotherapy had a notable positive impact in reducing the enucleation rate within the Leukocoria subgroup, but no significant effect was observed within the strabismus subgroup. Even with the addition of local chemotherapy to the treatment regimen, the enucleation rate remained unaffected in this particular subgroup. The possible explanation for this finding could be that Rb patients who primarily presenting with strabismus, generally have smaller tumors at lower clinical stages than those with Leukocoria, owing to involvement of the posterior pole and earlier detection of their malignancy. It is important to highlight that despite the lack of response to chemotherapy in terms of globe salvage, patients in the strabismus subgroup still exhibited a significantly lower overall enucleation rate compared to the Leukocoria subgroup after receiving systemic and local chemotherapy (76% vs 44%, Fig. 2c) and almost equal to general rate of enucleation in Rb patients (44% vs. 45%). These findings emphasize the critical importance of early detection of Rb; even when systemic or local chemotherapy are not available as treatment options, conventional treatment methods can successfully salvage the globe when Rb is timely detected. However, as is depicted in Fig. 4b, administering systemic chemotherapy to these patients proves to have a significant impact on reducing the mortality rate. Based on our findings’, targeted chemotherapy has less impact on reducing the mortality and enucleation rate in Rb patients with strabismus as their primary presentation. Further studies are needed to investigate whether this finding can be generalized to all Rb patients with this initial presentation. Our results showed it indicates that currently available treatment options are unable to salvage the eyes of nearly half of the Rb patients. This finding justifies exploring more effective treatment options such as molecularly targeted therapies, gene therapy, and innovative drug delivery methods [41]. 

The signs and symptoms of Rb depend on its size and location. There is a wide variation in the clinical presentation of children affected by retinoblastoma, worldwide [31, 44]. Leukocoria (which is seen when the tumor is large in size) is the most frequent presenting sign of Rb, found in approximately 60–70% of cases, followed by strabismus (10–20%) and proptosis (7%). Although LMICs and HICs do not differ in terms of common presenting sign, the frequency of advanced disease related signs like cellulitis and red eye and proptosis are more common in LMICs [31]. In addition, extraocular extension of retinoblastoma and even distant metastasis is highly prevalent in LICs [45]. The presenting signs of Iranian patients are similar to the global picture in terms of leukocoria and strabismus whereas, redness and painful eye is the third most common sign followed by proptosis [25, 26]. Furthermore, our new data from Iran showed that the frequency of eyes with redness were significantly reduced from 10 to 3% in recent years implying a trend of reduction of advanced cases [22]. At the diagnostic time, 50% and 20% of the patients in LMICs have extraocular R and metastasis, respectively. On the other hand, the incidence of extraocular involvement and metastasis remain less than 2% in high-income countries [31]. Our first report showed that 51% of Iranian patients presented with extraocular extension during 1990–2001 decreasing to 2% during 2001–2018 based on our latest report [9, 25]. 

The key limitation of this study was the lack of available data regarding the stage of retinoblastoma at the time of diagnosis at T1 period. Due to a significant amount of missing data for the classification of tumors in this period(T1), we could not evaluate the correlation between disease severity and response to treatment precisely. Additionally, it could be argued that the higher mortality and morbidity rates observed in patients during the T1 and T2 time periods were influenced by the longer follow-up duration for these patients. To mitigate this potential bias, we implemented a minimum follow-up period of 5 years for this study. However, it is important to acknowledge that the possibility of this bias could not be completely eliminated. Finally, we were not able to incorporate the data about the cause of death during the first part of our study; Whether the mortality is related to either the metastasis or the side effects of external beam radiotherapy/systemic chemotherapy.

## Conclusion

In conclusion, this study revealed that of the new era of systemic chemotherapy led to a noticeable decrease in mortality and morbidity rates in a developing country. Furthermore, our findings indicate that although adding targeted chemotherapy to systemic treatment did not result in higher survival rates, it did significantly reduce the need for enucleation. Finally, finding more effective treatment options with fewer side effects can reduce the burden of the disease and enhance the quality of life in patients with retinoblastoma.

## Data Availability

No datasets were generated or analysed during the current study.

## Abbreviations

Rb: Retinoblastoma
VEC: Vincristine, Etoposide and Carboplatin
IAC: Intra-artrial chemotherapy
SEER: Surveillance – Epidemiology and Ende Results
ICRB: International classification of Retinoblastoma
LMICs: Low to Middle income Countries
HICs: High income Countries
